# i stands for

**DOI:** 10.1016/j.isci.2018.03.001

**Published:** 2018-03-23

**Authors:** Stefano Tonzani

**Affiliations:** Lead Editor, *iScience*

## Main Text

The title of an Editorial does not typically start with a lowercase letter, so this column, which opens the first issue of *iScience*, is the exception rather than the rule. As editors, we will work to make the journal itself the exception in many different ways.

*iScience*, the newest open access journal from Cell Press, aims to address two needs within the research community: creating connections (between scientific topics and scientists of different fields) and meeting researchers’ requirements for the publishing process. To realize these aims, we are creating a new journal with several key characteristics, many of which start with the letter “i”—hence the journal name.

The “i” word that is central to our vision for the journal is **interdisciplinary**. We acknowledge upfront that not all research can (or needs to) be interdisciplinary; to be published in *iScience*, it is not a requirement for a paper to straddle two or more fields. At the same time, interdisciplinary research is clearly of increasing interest across the research enterprise, so we aim to create a forum that connects different communities and to seek out and highlight work that crosses boundaries. Obtaining funding for interdisciplinary proposals, getting people from different fields to speak the same language when performing research, and navigating an often-fraught publication process have all been pointed out as obstacles for interdisciplinary research. We aim to help in several ways. To begin with, many members of our editorial board run highly interdisciplinary labs, so they know firsthand the value and challenges of research that crosses boundaries. Our editors strive to provide a fair peer review process, in which the various expertise areas needed to evaluate a paper are accurately represented. Our editors, for example, often note to referees which other fields of expertise will complement their own in the manuscript evaluation process. The *iScience* editors also strive to reconcile different points of view among referees (for example, using reviewer discussion fora); this is critical for interdisciplinary manuscripts, as the total is often more than the sum of its parts.

The editors of *iScience* (in collaboration with our editorial and advisory boards, as well as peer reviewers) are empowered to select manuscripts that advance their fields—that is, papers that are **important** to publish and share. *iScience* will be a selective venue and will showcase inspiring research across its entire scope, covering the life, physical, and earth sciences.

In addition to building connections, *iScience* aims to tear down walls so as to be more **inclusive**. *iScience* is a Cell Press journal not just in the sense that it is published by Cell Press, but also because it builds on the collective expertise of all Cell Press editors, as well as the communities served by their journals. Cell Press editors inform our decisions: for example, they can pre-accept directly into *iScience* (in selected situations and in consultation with the *iScience* editors) papers that advance their field and have robust methods and data. This collaborative ethos, and the effort we put into making decisions based on what is already reported in the paper, allows *iScience* to be more **immediate**, bringing quickly to the community a story that is complete and interesting on its own. The scientific community can then build upon those results, for example by providing more mechanistic details.

At the core of what Cell Press does, there is a passion for science and a desire to help scientists, which includes a deep commitment to **integrity**. Along these lines, *iScience* will strive to remove unnecessary obstacles toward reproducibility of the data shown in the manuscripts it publishes. For example, our “Transparent Methods” section will include provisions to avoid citations of previous publications as a substitute for providing the details of a procedure. We encourage data deposition in open repositories whenever possible. It is a condition for publication in *iScience*, as for all Cell Press journals, that authors must be willing to distribute materials and protocols to qualified researchers, with minimal restrictions and in a timely manner.

We envision *iScience* as a platform that will evolve like the inspiring science it wants to publish. We do not claim to have all the answers today; however, together with the help of our scientific community, we will work hard to find them.

